# The Relationship Between Negative Life Events and Internalizing Problems: The Mediating Role of Self-Esteem and the Moderating Role of Resilience

**DOI:** 10.3390/bs16060845

**Published:** 2026-05-25

**Authors:** Dexian He, Jiaxin Mai, Xianyou He

**Affiliations:** 1 Department of Psychology, Guangdong University of Education, Guangzhou 510631, China; dexianhe@m.scnu.edu.cn (D.H.);; 2 Guangdong Key Laboratory of Mental Health and Cognitive Science, South China Normal University, Guangzhou 510631, China; 3 Center for Studies of Psychological Application, South China Normal University, Guangzhou 510631, China; 4 School of Psychology, South China Normal University, Guangzhou 510631, China

**Keywords:** negative life events, internalizing problems, self-esteem, resilience, moderated mediation

## Abstract

Negative life events (NLEs) are robust environmental correlates of adolescent internalizing problems (IPs), yet the psychological mechanism and boundary conditions remain unclear. To examine whether self-esteem accounts for the association between NLEs and adolescent IPs, and whether resilience conditions these links, 400 adolescents completed anonymous measures assessing NLEs, IPs, self-esteem, and resilience. The results show that (1) NLEs were positively associated with IPs, (2) self-esteem mediated the association between NLEs and IPs, (3) resilience moderated the relationship between NLEs and IPs, and (4) resilience also moderated the link between NLEs and self-esteem, such that associations were weaker at higher resilience. The index of moderated mediation was significant, indicating that the indirect effect via self-esteem decreased as resilience increased. These findings suggest that reduced self-esteem is an important link between exposure to negative life events and internalizing symptoms. Moreover, the findings indicate that resilience functions as a protective factor that attenuates both direct and indirect associations, suggesting potential targets for school-based prevention aimed at strengthening self-worth and resilience.

## 1. Introduction

Adolescence represents a critical developmental period characterized by rapid neurobiological maturation and shifting social roles, rendering individuals particularly vulnerable to psychological maladjustment (Blakemore & Mills, 2014; Casey et al., 2008; Silk et al., 2012). Among these challenges, internalizing problems, including depression, anxiety, social withdrawal, and somatic complaints, have emerged as a major global public health concern affecting a substantial proportion of youth worldwide (Achenbach & Edelbrock, 1981; Patel et al., 2007; Racine et al., 2021). Unlike externalizing behaviors, internalizing symptoms are often covert and emotionally inhibited, which increases the likelihood that they remain unrecognized by parents and educators (McLeod et al., 2007).

Accumulating evidence identifies negative life events (NLEs), such as academic failure, interpersonal conflict, and family disruptions, as salient environmental antecedents of adolescent internalizing problems (Hazzard et al., 2023; Liu et al., 1997). Longitudinal studies indicate that internalizing distress triggered by such stressors not only undermines immediate academic and social functioning but also confers long-term risk for psychiatric disorders and maladaptive outcomes in adulthood (Clayborne et al., 2019; Essau et al., 2014). Given the rising prevalence and lasting consequences of adolescent internalizing problems, identifying the psychological mechanisms and boundary conditions through which negative life events exert their effects is of substantial theoretical and practical significance.

Grounded in the Risk–Protective Factor framework (Rutter, 1987), this study conceptualizes the association between negative life events and adolescent internalizing problems as a dynamic process shaped by both vulnerability mechanisms and protective resources. Specifically, we propose a moderated mediation model in which self-esteem operates as an important psychological mechanism linking negative life events to internalizing problems, while psychological resilience functions as a boundary condition that modulates both direct and indirect associations. Within this framework, the Diathesis–Stress Model (Monroe & Simons, 1991) and resilience perspectives (Masten, 2001) are integrated as complementary accounts. The former specifies the vulnerability pathway through which adversity erodes psychological resources, whereas the latter delineates the protective resources that may attenuate this pathway.

### 1.1. Negative Life Events and Adolescent Internalizing Problems

The Diathesis–Stress Model posits that psychopathological symptoms arise from the interaction between an individual’s internal predisposition and external environmental stressors (Monroe & Simons, 1991). Negative life events (NLEs), defined as diverse socio-psychological stressors such as academic pressure, interpersonal conflicts, and experiences of loss, are closely linked to adolescent psychological distress (Liu et al., 1997). Empirical studies consistently demonstrate that frequent exposure to NLEs is associated with higher levels of internalizing symptoms, including anxiety and depression, across adolescence and emerging adulthood (Duprey et al., 2018; Gao et al., 2019; Jenness et al., 2019; Ruttle et al., 2014; Xia et al., 2022; Zhou et al., 2024). When adolescents encounter persistent negative feedback from their environment, they often struggle with emotional dysregulation. This dysregulation may, in turn, manifest as internalized distress.

The literature presents two competing theoretical perspectives on how exposure to NLEs influences psychological functioning. One perspective, derived from steeling and inoculation frameworks, posits that exposure to moderate levels of adverse life events may promote the development of coping capacities and psychological strengths. These capacities may reduce the likelihood of maladaptive responses to later stressors (Rutter, 1987; Seery et al., 2010). From this view, repeated but manageable challenges may contribute to adaptive stress regulation and lower internalizing symptoms over time. In contrast, the stress sensitization framework argues that prior exposure to aversive experiences lowers individuals’ threshold for subsequent stress responses, increasing vulnerability to affective dysregulation and internalizing psychopathology when encountering later life events (McLaughlin et al., 2010; Monroe & Harkness, 2005). According to this model, early and frequent NLEs may sensitize emotion-regulatory systems, such that even normative stressors elicit stronger psychological distress.

Empirical evidence from adolescent and emerging adult samples more consistently supports the sensitization perspective. Longitudinal studies show that cumulative negative life events are associated with greater increases in depressive and anxiety symptoms over time, even after accounting for prior symptom levels (Hammen et al., 2012; McLaughlin et al., 2010). Moreover, moderate adversities do not appear to confer protective effects against future internalizing symptoms when early life stress is high or chronic (Grant et al., 2003; McLaughlin & Nolen-Hoeksema, 2011).

Thus, consistent with stress sensitization theory and prior empirical work, we propose

**Hypothesis** **1.** *Negative life events are significantly and positively associated with internalizing problems in adolescents.*

### 1.2. The Mediating Role of Self-Esteem

Self-esteem is a core self-evaluative resource reflecting individuals’ global sense of self-worth (Rosenberg, 1965). Developmental evidence suggests that self-esteem is not only relatively stable but also systematically shaped by life experiences, especially events that threaten social belonging, competence, or role functioning—features that are highly salient during adolescence (Reitz, 2022). Accordingly, frequent exposure to NLEs (e.g., interpersonal rejection, academic setbacks, family disruptions) may undermine adolescents’ self-worth, thereby creating a psychological vulnerability that increases the likelihood of internalizing distress.

Consistent with vulnerability models, a meta-analysis of longitudinal studies showed that low self-esteem was prospectively associated with later depression more strongly than depression was associated with subsequent self-esteem decline, providing robust evidence that self-esteem functions as an antecedent risk factor rather than merely a consequence of internalizing symptoms (Sowislo & Orth, 2013). Furthermore, Steiger et al. (2014) demonstrated that both low baseline self-esteem and declines in self-esteem during adolescence significantly predicted depressive symptoms in adulthood. Complementing these findings, longitudinal work that separates stable between-person differences from within-person fluctuations indicates that periods of lower-than-usual self-esteem are followed by increases in depressive symptoms among adolescents. This evidence further supports a temporal ordering from self-esteem to internalizing problems (Masselink et al., 2018). In clinical samples, high self-esteem at baseline has been found to predict reductions in symptoms of both anxiety and depression over a three-year follow-up, supporting self-esteem as a protective factor against emotional difficulties (Henriksen et al., 2017). Recent meta-analytic reviews also conclude that self-esteem prospectively predicts both depression and anxiety across the lifespan, even when prior symptoms and relevant confounds are controlled. These findings underscore self-esteem as a plausible mechanism through which adversity contributes to internalizing psychopathology (Orth & Robins, 2022). Together, these findings provide support for conceptualizing self-esteem as a central mechanism through which early exposure to NLEs may be linked to mental health.

Emerging empirical studies further suggest that self-esteem can serve as a statistical pathway transmitting the effects of adverse experiences on adjustment outcomes. For instance, Quan and Sun (2024) found that self-esteem mediated the association between negative life events and college students’ adjustment, with the indirect effect varying by academic grade, highlighting the role of self-esteem in stress adaptation processes. Among adolescent samples, Kang et al. (2024) similarly reported that self-esteem mediated the link between negative parenting style and depression, supporting self-esteem as a mechanism connecting adverse family contexts to internalizing outcomes. Beyond its mediating role, self-esteem may also function as a protective factor. Yang et al. (2023) demonstrated in a large sample of Chinese college students (*N* = 6224) that self-esteem moderated the association between negative life events and both trait anxiety and depression. Specifically, these associations were weaker among students with higher self-esteem.

Despite this growing body of evidence, important gaps remain that this study seeks to address. First, existing mediation evidence varies substantially in population characteristics (college students vs. adolescents), the operationalization of adversity (e.g., parenting context vs. broader NLE exposure), and the scope of outcome measures. Moreover, prior research has often focused on relatively narrow outcomes, especially depressive symptoms. However, adolescents’ emotional maladjustment frequently presents in broader and overlapping forms. By operationalizing the outcome variable as a multifaceted construct of internalizing problems rather than focusing on depression, this study aims to provide a more comprehensive account of adolescents’ emotional difficulties associated with negative life events. While these findings point to self-esteem as a potential mediating mechanism, it remains unclear whether this mediating role extends to adolescents’ broader internalizing problems in the context of general negative life event exposure.

Second, although prior studies have separately examined the mediating role of self-esteem (e.g., Kang et al., 2024; Quan & Sun, 2024) and the moderating role of resilience (e.g., Anyan & Hjemdal, 2016; Wei et al., 2022) in stress-related pathways, few have integrated these mechanisms within a single conditional process framework. Existing moderated mediation studies have predominantly focused on resilience as a moderator of the direct path from stress to psychopathology, while relatively neglecting whether resilience also conditions the first-stage path through which adversity undermines self-evaluative resources. Anyan et al. (2018) demonstrated that resilience moderated the indirect effects of specific stress dimensions on depressive symptoms, noting that “resilience does not only buffer direct, but also indirect psychological adversities” (p. 72). However, these authors focused on anxiety as the mediator rather than self-esteem, leaving the question of whether resilience protects the self-system pathway unexamined. Given that not all adolescents exposed to NLEs exhibit self-esteem erosion or internalizing symptoms to the same extent, integrating self-esteem mediation within a conditional process framework that considers resilience as a boundary condition may yield a more precise account of when this pathway is most pronounced.

Based on these considerations, we propose

**Hypothesis** **2.** *Self-esteem mediates the association between negative life events and internalizing problems.*

### 1.3. The Moderating Role of Resilience

While exposure to negative life events (NLEs) constitutes a pervasive risk factor, adolescents vary considerably in their psychological responses. The association between NLEs and internalizing problems may therefore depend on protective conditions. Resilience is commonly conceptualized as a dynamic protective process through which promotive resources buffer the psychological impact of adversity exposure and reduce vulnerability to maladaptive outcomes (Luthar et al., 2000; Masten, 2001, 2014; Zimmerman et al., 2013). Resilience frameworks further distinguish compensatory processes, in which promotive resources exert direct beneficial effects on adjustment, from protective processes, in which such resources interact with risk exposure to attenuate its adverse consequences (Fergus & Zimmerman, 2005; Rutter, 1987). This protective factor perspective provides a theoretical basis for treating resilience as a boundary condition that shapes the strength of the association between NLEs and internalizing problems.

Mechanistically, resilience is thought to operate partly through more effective regulatory processes, including adaptive appraisal and meaning-making in the face of adversity (Tugade & Fredrickson, 2004). Given that emotion dysregulation is a well-established correlate of adolescent anxiety and depression, regulatory capacities associated with resilience represent plausible pathways through which resilience may attenuate internalizing risk under heightened NLE exposure (Young et al., 2019). Empirical evidence is consistent with this buffering account in adolescents. For example, protective factors associated with resilience have been shown to weaken the association between life events and adolescent depressive symptoms (Askeland et al., 2020). Similarly, among Chinese adolescents, resilience buffered the link between stressful life events and depression and weakened downstream risk processes in a conditional model (Wei et al., 2022). Anyan and Hjemdal (2016) further demonstrated that resilience moderated both mediated and direct pathways linking stress to symptoms of anxiety and depression among Norwegian adolescents. This finding highlights its potential to attenuate risk at multiple points in the stress–distress process.

In the context of the present model, resilience is expected to play a dual moderating role. First, resilience may weaken the direct association between NLEs and internalizing problems by strengthening coping resources and stress-buffering processes. In this way, resilience may reduce the psychological impact of adverse experiences (S. Cohen & Wills, 1985; Zimmerman et al., 2013). Second, and more novel to the present investigation, resilience may buffer the extent to which NLEs undermine the self-system. Adolescents’ self-esteem responses to negative events depend partly on how such experiences are appraised and interpreted. Cognitive vulnerability work indicates that maladaptive attributional styles can amplify affective reactions to negative events (Calvete et al., 2008). Furthermore, self-esteem-related vulnerability to life stressors is shaped in part by cognitive interpretations of adverse experiences (Southall & Roberts, 2002). From this perspective, resilience-related capacities (e.g., adaptive appraisal and meaning-making) may help adolescents maintain self-worth under NLE exposure (Tugade & Fredrickson, 2004). By contrast, lower resilience may be associated with stronger self-esteem erosion, thereby increasing the likelihood of downstream internalizing distress. Although prior research has examined resilience as a moderator of the direct stress–outcome pathway, relatively few studies have tested whether resilience also conditions the first-stage path through which adversity undermines self-evaluative resources. This gap is noteworthy because, if resilience buffers the NLEs–self-esteem link, the indirect pathway to internalizing problems should be attenuated, or even become non-significant, at higher levels of resilience.

Therefore, we propose

**Hypothesis** **3.** *Resilience moderates the association between negative life events and internalizing problems, such that this association is weaker at higher levels of resilience.*

We further propose

**Hypothesis** **4.** *Resilience moderates the association between negative life events and self-esteem, such that the negative association between negative life events and self-esteem is weaker at higher levels of resilience.*

### 1.4. This Study

This study proposes a moderated mediation model linking negative life events to adolescent internalizing problems, thereby extending prior work in several ways. First, this study conceptualizes internalizing problems as a multifaceted construct rather than limiting the outcome to depressive symptoms alone. Adolescents’ internalizing difficulties often involve co-occurring manifestations, including anxious/depressed mood, social withdrawal, and somatic complaints (Achenbach & Edelbrock, 1981). By adopting this broader operationalization consistent with the multidimensional nature of internalizing psychopathology (Krueger, 1999; Watson, 2005), this study aims to provide a more comprehensive understanding of how negative life events are associated with adolescent emotional maladjustment.

Second, this study integrates self-esteem mediation and resilience moderation within a unified conditional process framework. Previous research has examined these mechanisms largely in isolation. Self-esteem has been investigated as a mediator between adversity and psychopathology (Sowislo & Orth, 2013; Zhao et al., 2023), whereas resilience has been examined as a moderator of stress–outcome relationships (Anyan & Hjemdal, 2016; Salami, 2010). To our knowledge, no study has simultaneously tested whether resilience moderates both the direct association and the first-stage indirect pathway through self-esteem in adolescent samples. This first-stage moderated mediation approach (Edwards & Lambert, 2007) allows for a more precise identification of the boundary conditions under which the self-esteem mechanism is operative. Specifically, if resilience buffers the extent to which NLEs erode self-esteem, the indirect pathway to internalizing problems should be attenuated at higher levels of resilience. However, this specific hypothesis has yet to be empirically tested in adolescent samples using a comprehensive moderated mediation framework.

**Figure 1:**
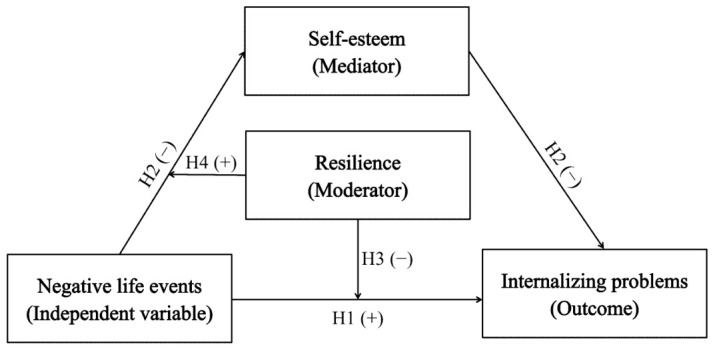
Proposed model of the relationship between negative life events and adolescent internalizing problems.

Based on previous studies, we propose the following hypotheses: (1) Negative life events are significantly and positively associated with internalizing problems in adolescents. (2) Self-esteem mediates the association between negative life events and adolescent internalizing problems. Specifically, negative life events are negatively associated with self-esteem, and self-esteem is negatively associated with internalizing problems. (3) Resilience moderates the association between negative life events and internalizing problems. (4) Resilience moderates the association between negative life events and self-esteem. The conceptual model is shown in Figure 1.

## 2. Materials and Methods

### 2.1. Participants

The participants in this study were recruited from public middle schools in Guangzhou, China, using a convenience sampling method. Questionnaires were administered during class following ethical protocols. The researchers explained the purpose and procedures of the study, emphasizing that participation was voluntary and that participants could withdraw at any time without penalty. After obtaining informed consent, 427 questionnaires were distributed. Data were excluded for 27 participants due to incomplete responses or failing more than two of three attention catch trials embedded throughout the survey. The final sample consisted of *N* = 400 participants, corresponding to a valid response rate of 93.7%. The sample comprised 196 males (49.0%) and 204 females (51.0%). The participants ranged in age from 14 to 19 years, with a mean age of 15.98 years (*SD* = 0.78). Data collection was conducted between January and March 2025. This adolescent sample was selected specifically because middle school represents a critical stage of identity formation and heightened academic pressure, during which individuals are particularly susceptible to internalizing psychological distress.

An a priori power analysis was conducted using G*Power 3.1.9.6. Based on the effect sizes reported in recent similar research (Ye et al., 2025), we targeted a conservative small-to-medium effect size (*f*^2^ = 0.05). With an alpha level of 0.05 and 80% statistical power, a sample size of approximately 278 participants was required. Our final sample of 400 participants provides sufficient power to detect both the mediation and moderation effects within the proposed model.

### 2.2. Measurements

#### 2.2.1. Negative Life Events

Adolescent exposure to stressors was assessed using the Adolescent Self-Rating Life Events Checklist (ASLEC), originally developed by Liu et al. (1997). This 27-item instrument evaluates the frequency and perceived impact of negative life events across six domains: interpersonal relationships, academic pressure, punishment, loss, health and adaptation, and other stressors. Participants indicated the impact of events occurring within the past 12 months on a 5-point Likert scale, ranging from 0 (no impact) to 4 (extremely severe). Higher total scores reflect a greater accumulation of negative life stress. In this study, the scale demonstrated excellent internal consistency, with Cronbach’s α = 0.89.

#### 2.2.2. Internalizing Problems

We employed the Anxious/Depressed subscale from the Youth Self-Report (YSR), a widely recognized tool for assessing emotional distress (Achenbach, 1991), to measure adolescent internalizing problems. The subscale consists of 13 items that capture symptoms such as persistent crying, fear, and loneliness. Each item is scored on a 3-point scale: 0 (not true), 1 (somewhat or sometimes true), and 2 (very true or often true). Higher scores indicate a higher severity of internalizing symptoms. Cronbach’s α for this subscale in the current sample was 0.89.

#### 2.2.3. Self-Esteem

Individual self-worth was evaluated using the Self-Esteem Scale (SES) developed by Rosenberg (1965), specifically utilizing the Chinese version translated and validated by Wang et al. (1999). This unidimensional scale comprises 10 items rated on a 4-point Likert scale from 1 (strongly disagree) to 4 (strongly agree). After reverse-scoring appropriate items, higher mean scores represent more positive self-evaluations. The SES has shown high reliability in previous Chinese adolescent samples, and its Cronbach’s α in this study was 0.85.

#### 2.2.4. Psychological Resilience

Adolescents’ resilience was measured via the Connor–Davidson Resilience Scale (CD-RISC; Connor & Davidson, 2003), using the Chinese adaptation refined by Yu and Zhang (2007). The 25-item scale encompasses three dimensions: tenacity, strength, and optimism. Participants responded on a 5-point Likert scale (0 = not true at all to 4 = true nearly all the time), with higher scores indicating greater psychological resilience. In our study, the scale exhibited robust reliability, with Cronbach’s α = 0.93.

### 2.3. Data Analysis

Data analysis was performed using IBM SPSS Statistics 27.0 (IBM Corp., Armonk, NY, USA). First, descriptive statistics and Pearson correlation coefficients were calculated to examine the relationships among negative life events, internalizing problems, self-esteem, and resilience. Given that the data were collected via self-report measures, the potential for common method bias was evaluated using multiple approaches before hypothesis testing, including Harman’s single-factor test and confirmatory factor analysis (CFA).

The PROCESS macro (Version 4.0) for SPSS (Hayes, 2022) was used to test the proposed research hypotheses. Specifically, Model 4 was used to examine the mediating role of self-esteem in the relationship between negative life events and internalizing problems. Subsequently, Model 8 was applied to test the moderated mediation model, assessing whether resilience moderated both the direct association between negative life events and internalizing problems and the indirect path via self-esteem. The significance of the indirect effects and conditional indirect effects was evaluated using the bias-corrected bootstrap method with 5000 resamples to generate 95% confidence intervals (CIs). In all regression analyses, gender and age were included as covariates to account for their potential influence on the outcome variables. Although age did not reach statistical significance in any model (all *p values* > 0.05), it was retained to ensure demographic comprehensiveness, as age is considered an important control variable in adolescent research.

## 3. Results

### 3.1. Common Method Bias Test and Multicollinearity Diagnostics

Given that all study variables were assessed via self-report measures, the potential for common method bias was evaluated using multiple approaches (Podsakoff et al., 2003). Harman’s single-factor test indicated that the first unrotated factor accounted for 22.10% of the total variance, below the conventional 40% threshold. To evaluate this concern more rigorously, we conducted a confirmatory factor analysis (CFA) using the lavaan package in R 4.3.2 (R Core Team, 2023; Rosseel, 2012), employing item parceling with three parcels per construct (Little et al., 2002). The hypothesized four-factor model demonstrated acceptable fit (χ^2^(48) = 169.19, CFI = 0.955, TLI = 0.938, RMSEA = 0.079, and SRMR = 0.067) and fit significantly better than a single-factor model (Satorra–Bentler scaled Δχ^2^(6) = 4742, *p* < 0.001; χ^2^(54) = 1134.54, CFI = 0.599, and RMSEA = 0.224). The AVE values for all constructs exceeded 0.50 (Fornell & Larcker, 1981), and the squared inter-factor correlations did not exceed the AVE of either factor. Taken together, the results of Harman’s single-factor test and the CFA model comparison provide converging evidence that common method bias is unlikely to pose a serious threat to the validity of the present findings. They also confirm that the four study constructs are empirically distinct. In addition, all variance inflation factors (VIFs) were below 5, well within the acceptable threshold (Hair et al., 2019), suggesting that multicollinearity was not a concern in the regression analyses.

### 3.2. Descriptive Statistics and Correlation Analysis

The descriptive statistics and Pearson correlation coefficients for the main variables are presented in Table 1. As expected, NLEs were significantly and positively correlated with internalizing problems (IPs) (*r* = 0.61; *p* < 0.01). Conversely, NLEs showed significant negative correlations with both self-esteem (*r* = −0.37; *p* < 0.01) and resilience (*r* = −0.25; *p* < 0.01). Furthermore, IPs were negatively correlated with self-esteem (*r* = −0.54; *p* < 0.01) and resilience (*r* = −0.39; *p* < 0.01), while self-esteem was positively correlated with resilience (*r* = 0.62; *p* < 0.01).

### 3.3. Testing for the Mediating Role of Self-Esteem

**Figure 2:**
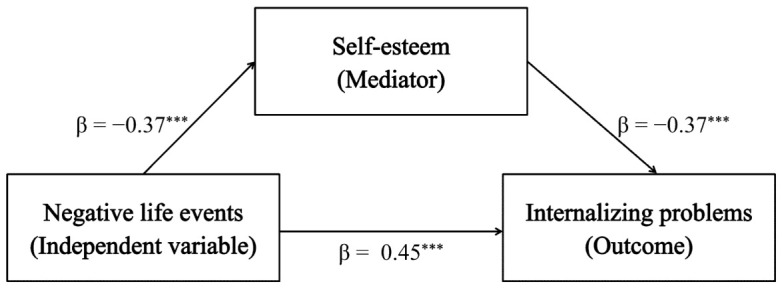
The mediating role of self-esteem between negative life events and internalizing problems. *** *p* < 0.001.

To examine the statistical mediating role of self-esteem in the relationship between NLEs and IPs, we conducted a mediation analysis using Model 4 of the PROCESS macro for SPSS (Hayes, 2022), controlling for gender and age. The results showed that NLEs were positively associated with IPs (β = 0.58, *SE* = 0.02, *t* = 14.65, *p* < 0.001, *R*^2^ = 0.39, *F* = 85.00, and *p* < 0.001) (path c). NLEs were significantly negatively associated with self-esteem (β = −0.37, *SE* = 0.02, *t* = −7.76, *p* < 0.001, *R*^2^ = 0.13, *F* = 20.54, and *p* < 0.001) (path a). When both NLEs and self-esteem were entered into the regression model, self-esteem was significantly negatively associated with IPs (β = −0.37, *SE* = 0.04, *t* = −9.83, *p* < 0.001, *R*^2^ = 0.51, *F* = 103.29, and *p* < 0.001) (path b). The direct association of NLEs with IPs remained significant (β = 0.45, *SE* = 0.02, *t* = 11.63, and *p* < 0.001) (path c′). These findings are consistent with a mediational process in which self-esteem partially accounts for the association between NLEs and IPs. In terms of effect size, the model accounted for a moderate proportion of variance in self-esteem (*R*^2^ = 0.13) and a large proportion of variance in internalizing problems (*R*^2^ = 0.51), according to conventional standards (J. Cohen, 1988). In addition, the indirect effect via self-esteem accounted for approximately 23.5% of the total association, suggesting that self-esteem represents an important, though partial, explanatory pathway linking NLEs to IPs (see Figure 2 and Table 2).

### 3.4. Testing the Moderated Mediation Effect

**Figure 3:**
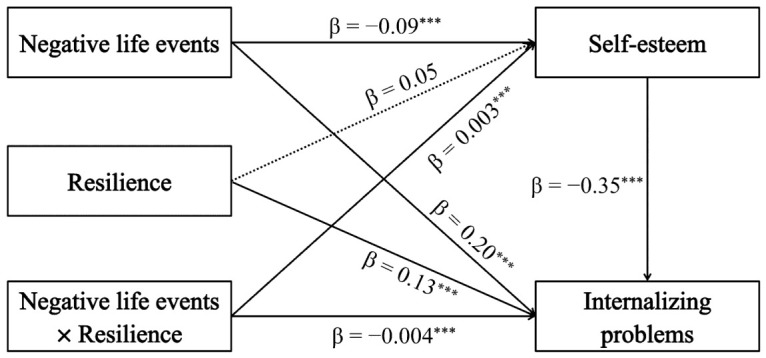
Model of the moderating role of resilience in the direct and indirect relationship between negative life events and internalizing problems. *** *p* < 0.001.

To test Hypotheses 3 and 4, we used PROCESS Model 8 to examine whether resilience moderated the direct association between negative life events (NLEs) and internalizing problems (IPs) and the first-stage association between NLEs and self-esteem, while controlling for gender and age. As shown in Table 3, the interaction between NLEs and resilience significantly predicted self-esteem (β = 0.003, *t* = 3.37, *p* < 0.001, and 95% CI [0.001, 0.005]) and internalizing problems (β = −0.004, *t* = −4.01, *p* < 0.001, and 95% CI [−0.006, −0.002]). The interaction terms accounted for an additional 2% of the variance in each respective model (Δ*R*^2^ = 0.02), indicating a modest increment in the explained variance (W. F. Chaplin, 1991) (see Figure 3 and Table 3).

**Figure 4:**
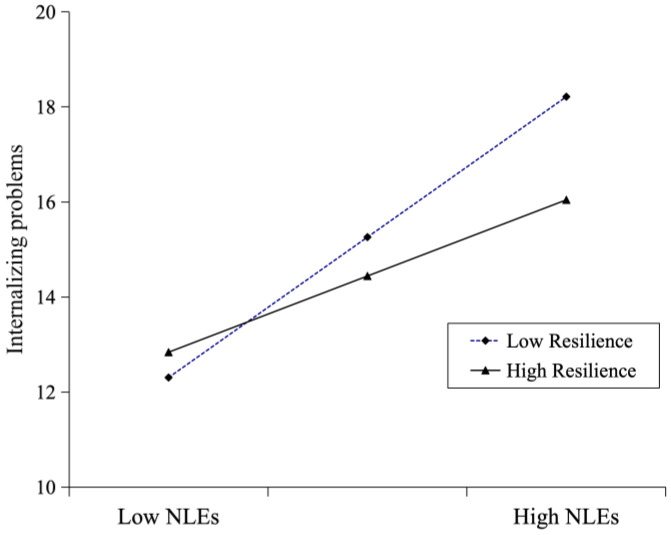
Simple slope analysis of resilience’s moderating effect between negative life events (NLEs) and internalizing problems.

Simple slope analyses were conducted to further interpret these moderating effects. As illustrated in Figure 4, for adolescents with low resilience (−1 SD), negative life events showed a stronger positive association with internalizing problems (β = 0.26, *SE* = 0.02, *t* = 11.32, *p* < 0.001, and 95% CI [0.212, 0.301]). For those with high resilience (+1 SD), this association remained significant but was attenuated (β = 0.14, *SE* = 0.02, *t* = 6.48, *p* < 0.001, and 95% CI [0.096, 0.180]). The practical significance of resilience as a moderator is further reflected in the simple slope comparisons. Specifically, the association between NLEs and IPs was nearly twice as strong at low resilience (0.26) compared with high resilience (0.14), indicating meaningful differentiation in vulnerability across resilience levels.

**Figure 5:**
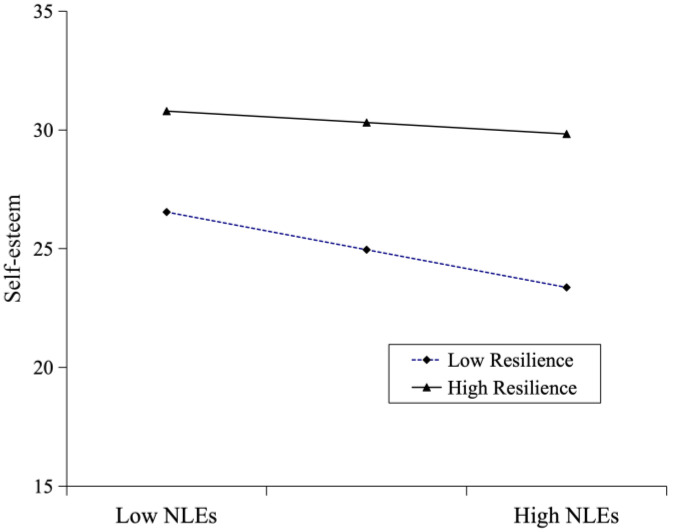
Simple slope analysis of resilience’s moderating effect between negative life events (NLEs) and self-esteem.

For the first-stage path, as shown in Figure 5, NLEs were more strongly negatively associated with self-esteem at low resilience (−1 SD; β = −0.14, *SE* = 0.02, *t* = −6.66, and *p* < 0.001) than at high resilience (+1 SD; β = −0.04, *SE* = 0.02, *t* = −2.14, and *p* < 0.05). This difference is also practically meaningful. At low resilience, the negative association between NLEs and self-esteem was more than three times stronger (−0.14) than at high resilience (−0.04), suggesting that resilience may play an important role in protecting adolescents’ self-esteem under adversity.

In addition, the index of moderated mediation was significant, index = −0.0011, Boot *SE* = 0.0004, and 95% CI [−0.0020, −0.0004]. This indicates that the indirect effect of NLEs on IPs via self-esteem significantly varied across levels of resilience and became weaker as resilience increased.

Finally, the conditional indirect effects are reported in Table 4. The indirect effect of NLEs on IPs via self-esteem was significant at low resilience (−1 *SD*) and at the mean level of resilience, but it became non-significant at high resilience (+1 *SD*). This pattern indicates that the self-esteem-mediated indirect association was progressively attenuated as resilience increased and was no longer statistically detectable at high levels of resilience.

## 4. Discussion

The present study tested a moderated mediation model to investigate the association between negative life events and internalizing problems among adolescents, as well as the underlying mechanisms involving self-esteem and resilience. It extended prior work in two ways: First, this study conceptualized internalizing problems as a multifaceted construct rather than limiting the outcome to depressive symptoms alone (e.g., Anyan & Hjemdal, 2016; Zhao et al., 2023). Second, this study integrated self-esteem mediation and resilience moderation within a unified conditional process framework. Prior research has examined these mechanisms separately (e.g., Anyan & Hjemdal, 2016; Sowislo & Orth, 2013; Wei et al., 2022). However, to our knowledge, no study has tested whether resilience moderates both the direct path and the first-stage indirect pathway through self-esteem in adolescent samples.

The findings support the proposed model. First, higher NLE exposure was robustly associated with greater internalizing problems. Second, self-esteem partially mediated this association, indicating that the association between NLEs and lower self-esteem may represent an important explanatory factor underlying the association between environmental adversity and internalizing distress. Third, resilience buffered the direct association between NLEs and internalizing problems, such that the positive NLEs–internalizing link was weaker at higher levels of resilience. Finally, resilience also moderated the first-stage path from NLEs to self-esteem, attenuating the negative association between NLEs and self-esteem. Consistent with this pattern, the conditional indirect effect via self-esteem was evident at low-to-average resilience but diminished and became non-significant at high resilience. Taken together, these findings support a risk-protective factor account (Rutter, 1987; Zimmerman et al., 2013) in which adverse life events increase internalizing problems partly through compromised self-worth. At the same time, resilience functions as a protective resource that stabilizes self-related processes and dampens risk pathways under adversity (Luthar et al., 2000; Masten, 2014).

### 4.1. The Direct Effect of Negative Life Events on Internalizing Problems

Consistent with Hypothesis 1, the present findings showed that negative life events were significantly and positively associated with adolescent internalizing problems. This finding aligns with the Diathesis–Stress Model (Monroe & Simons, 1991), which posits that psychopathological symptoms emerge when environmental stressors interact with an individual’s pre-existing vulnerabilities and exceed their coping resources (Colodro-Conde et al., 2018). In academically competitive contexts, adolescents may experience multiple, co-occurring stressors (e.g., academic setbacks, peer conflict, and family disruptions). The cumulative burden of such events may be linked to heightened anxiety, depressed mood, withdrawal, and somatic complaints.

Our findings are consistent with longitudinal studies demonstrating significant associations between negative life events and internalizing symptoms during adolescence. Exposure to stressful life events is a robust correlate of internalizing psychopathology, including depression and anxiety. Furthermore, adolescence involves higher levels of exposure to stressful life events. These experiences are more tightly coupled with increases in negative affect and psychopathology than in other developmental periods (Jenness et al., 2019).

The present results also align with the stress sensitization perspective, which proposes that repeated or chronic exposure to adversity can increase individuals’ sensitivity to subsequent stressors (McLaughlin et al., 2010; Monroe & Harkness, 2005). Under this hypothesis, exposure to childhood adversity lowers the threshold for tolerating future stressful events that trigger the onset of psychopathology or aggravate underlying vulnerabilities (McLaughlin et al., 2010). Prior adverse experiences may lower the threshold for affective and cognitive reactivity, making internalizing symptoms more likely under continued or even relatively minor subsequent challenges (Hammen et al., 2000; Stroud, 2019). Moreover, this sensitization effect appears to be stronger for stressful events that occur during adolescence compared with adulthood (Ruttle et al., 2014), highlighting the developmental significance of adolescence as a period of heightened vulnerability to stress. Taken together, the current evidence supports the conclusion that NLE exposure is a robust correlate of adolescent internalizing problems, consistent with prior findings linking stressful life events to depression and anxiety in youth populations (e.g., Duprey et al., 2017; Jenness et al., 2019; Ye et al., 2025).

The correlation between NLEs and IPs (*r* = 0.61) may raise concerns regarding shared method variance or conceptual overlap. Nevertheless, the association between NLEs and IPs is theoretically plausible and well established in the stress–psychopathology literature (Hammen, 2005; Jenness et al., 2019). Conceptually, the two constructs remain distinct: NLEs refer to externally experienced stressful events, whereas IPs reflect internal psychological symptoms (Achenbach & Edelbrock, 1981; Liu et al., 1997). This distinction was further supported by the CFA results reported in Section 3.1. Specifically, the hypothesized four-factor model fit the data substantially better than the single-factor model, providing evidence for the discriminant validity of the study constructs (Fornell & Larcker, 1981).

Female adolescents reported significantly higher levels of both negative life events and internalizing problems than males, consistent with prior research documenting greater vulnerability to internalizing disorders among adolescent girls (Milas et al., 2025; Zahn-Waxler et al., 2008). Two complementary frameworks help explain this pattern (Shih et al., 2006). The stress exposure model suggests that girls tend to experience higher levels of interpersonal stressors, given their greater investment in emotionally intimate relationships (Rose & Rudolph, 2006; Rudolph, 2002). The stress reactivity model further proposes that girls show stronger emotional responses to such stressors, as their self-concept is more closely tied to relationship quality. Gender socialization may further contribute, as girls are often socialized to be more relationally oriented and emotionally expressive than boys. This socialization potentially increases their sensitivity to interpersonal stressors and propensity to internalize negative affect (T. M. Chaplin & Aldao, 2013). Together, these factors offer a plausible account for the observed gender differences in the current sample.

### 4.2. The Mediating Role of Self-Esteem

Supporting Hypothesis 2, self-esteem partially mediated the association between negative life events and internalizing problems. This finding is consistent with vulnerability models, which conceptualize low self-esteem as a psychological diathesis associated with heightened risk for depressive and anxiety symptoms (Orth & Robins, 2022; Sowislo & Orth, 2013). Extending prior longitudinal evidence showing that low self-esteem prospectively predicts internalizing symptoms in both community and clinical samples (Henriksen et al., 2017; Masselink et al., 2018), our results suggest that one pathway through which adolescents’ NLE exposure may be linked to internalizing distress involves erosion of self-evaluative resources.

Several theoretical perspectives can be integrated to elucidate this statistical mediation effect. From a sociometer perspective, self-esteem functions as an internal gauge of social acceptance and relational value (Leary & Baumeister, 2000; Leary, 2005). Repeated exposure to adverse experiences, such as academic failure, peer rejection, or family conflict, may convey signals of social devaluation, thereby undermining adolescents’ global sense of self-worth. Once diminished, low self-esteem may activate negative self-schemas that bias information processing toward self-criticism, hopelessness, and anxiety, consistent with cognitive vulnerability theory (Beck, 1967; Haaga et al., 1991). Furthermore, from an anxiety-buffering perspective, self-esteem ordinarily serves as a psychological resource that mitigates the emotional impact of stress; when this resource is weakened, adolescents may become less capable of regulating affective responses to adversity (Greenberg et al., 1992; Pyszczynski et al., 2004). Integrating these perspectives within a diathesis–stress framework (Monroe & Simons, 1991), the present findings suggest that NLEs may function as environmental stressors associated with lower self-esteem. In turn, this lowered self-esteem may serve as a cognitive–affective vulnerability linked to greater internalizing distress. This integrated account positions self-esteem as a plausible pathway connecting environmental stress to psychological maladjustment.

The present findings extend prior mediation studies in several ways. First, whereas previous research has predominantly examined self-esteem as a mediator linking specific adversity types (e.g., negative parenting; Kang et al., 2024) to narrow outcomes (e.g., depressive symptoms), this study suggests that self-esteem may represent an important explanatory pathway linking broader NLE exposure to a multifaceted construct of internalizing problems. Second, the observed partial mediation suggests that while self-esteem erosion represents an important explanatory pathway, other mechanisms likely contribute to the NLEs–internalizing association independently of self-evaluative processes. Such mechanisms may include maladaptive coping strategies, deficits in social support, or direct physiological stress responses (Compas et al., 2017). Future research examining multiple mediators simultaneously may yield a more comprehensive account of how adversity is associated with psychological distress.

From an applied standpoint, these findings suggest that interventions targeting self-esteem preservation may help buffer adolescents against the internalizing consequences of negative life events. Cognitive restructuring strategies, which help adolescents disentangle specific failures from global self-evaluation, may be particularly effective in targeting this stress–distress pathway (Orth & Robins, 2022). School-based programs that target adolescents’ self-evaluative resources may similarly serve a protective function. For example, meta-analytic evidence indicates that self-concept interventions in educational settings are effective in enhancing students’ self-perceptions (O’Mara et al., 2006).

### 4.3. The Moderating Role of Resilience

Supporting Hypotheses 3 and 4, resilience emerged as a protective boundary condition that weakened both (a) the direct association between negative life events and internalizing problems and (b) the first-stage association between negative life events and self-esteem. These results align with risk–protective factor perspectives, which emphasize that promotive resources can interact with risk exposure to reduce the strength of maladaptive associations (Fergus & Zimmerman, 2005; Rutter, 1987; Zimmerman et al., 2013). From a developmental systems view, resilience reflects regulatory processes that help individuals adapt to stress across contexts rather than eliminating exposure (Lerner et al., 2023; Masten, 2001, 2014).

Regarding the direct path (NLEs → internalizing problems), simple slope analyses indicated that the positive association between NLE exposure and internalizing problems was weaker among adolescents with higher resilience. This pattern is consistent with buffering models, which propose that protective resources reduce the psychological impact of risk factors (S. Cohen & Wills, 1985). While some prior studies have conceptualized resilience as a mediator that may be depleted by stress (e.g., Geng et al., 2022; Ye et al., 2025), our findings align with recent empirical evidence supporting its role as a protective buffer that weakens the association between stressful life events and depressive symptoms (Anyan & Hjemdal, 2016; Wei et al., 2022). One plausible interpretation is that resilient adolescents may draw on stronger self-regulation and active coping resources, allowing them to maintain psychological stability despite exposure to environmental stressors. The practical significance of this moderating effect is reflected in the magnitude of the simple slope differences. Specifically, the association between NLEs and internalizing problems was nearly twice as strong among adolescents with low resilience compared with those with high resilience, indicating meaningful differentiation in psychological vulnerability across resilience levels.

Resilience also moderated the first stage of the mediation path (NLEs → self-esteem). This finding represents a key contribution of this study. Prior moderated mediation research has predominantly examined resilience as a moderator of the direct stress–outcome pathway (e.g., Anyan & Hjemdal, 2016; Wei et al., 2022). In contrast, relatively few studies have tested whether resilience also conditions the extent to which adversity undermines self-evaluative resources. The present results suggest that the negative association between NLEs and self-esteem was attenuated for adolescents with high resilience, whereas this association remained pronounced for those with low resilience. These findings are consistent with the Protective Factor Model, in which personal resources reduce the degree to which risk exposure undermines key developmental assets (Garmezy et al., 1984; Rutter, 1987). One possible interpretation is that resilience-related capacities, such as adaptive appraisal and meaning-making (Tugade & Fredrickson, 2004), may help adolescents maintain self-worth under adversity, preventing the erosion of self-esteem that would otherwise amplify internalizing risk. This suggests that resilience may operate not only by mitigating symptom severity directly but also by preserving the stability of the self-system against adverse environments. The practical significance of this effect is further evidenced by the simple slope comparisons: at low resilience, the negative association between NLEs and self-esteem was more than three times stronger than at high resilience, suggesting that resilience plays a particularly important protective role in safeguarding adolescents’ self-evaluative resources under conditions of adversity.

Concerning the conditional indirect effect, at low and average levels of resilience, the indirect pathway from NLEs to internalizing problems via self-esteem was statistically significant. This indicates that self-esteem erosion functioned as an explanatory mechanism linking adversity to psychological distress. However, at high levels of resilience, this indirect effect became non-significant. This pattern suggests that the self-esteem-mediated pathway may be substantially weakened at high levels of resilience. Consistent with Masten’s (2001) argument that resilience arises from the normative functioning of human adaptive systems, these findings indicate that adequate protective resources can buffer the impact of adversity, and that when such resources are compromised, developmental risk is substantially elevated. From a practical standpoint, this result implies that interventions aimed at bolstering resilience may reduce symptom severity directly while also protecting the self-system from adversity-related erosion, thereby disrupting a key mechanism through which negative life events contribute to internalizing problems.

This study extends prior moderated mediation research in several ways. First, whereas most prior studies have focused on resilience as a moderator of the direct stress–psychopathology link, this study provides evidence that resilience also buffers the first-stage path through which NLEs undermine self-esteem. This dual moderating role has rarely been examined within a unified conditional process framework in adolescent samples, particularly among Chinese adolescents. Second, the finding that the indirect effect via self-esteem becomes non-significant at high resilience levels offers further insight into the boundary conditions of the self-esteem mechanism. Rather than operating uniformly across all individuals, the self-esteem pathway appears to be most relevant for adolescents with fewer protective resources. This finding has important implications for targeted intervention efforts. Third, by examining self-esteem mediation and resilience moderation within a single model, this study provides a more integrated perspective on how negative life events are associated with internalizing problems. Furthermore, it clarifies how protective resources may attenuate this association.

Overall, these findings suggest that resilience is not only associated with lower internalizing problems directly. It may also reduce the strength of both the direct risk pathway from negative life events and the self-esteem-related pathway through which adversity is linked to internalizing symptoms. This dual protective function underscores the importance of resilience as a multifaceted resource that operates at multiple points in the stress–distress process.

### 4.4. Theoretical and Practical Implications

#### 4.4.1. Theoretical Implications

This study contributes to the literature by integrating diathesis–stress perspectives on environmental adversity and psychopathology (Monroe & Simons, 1991) with risk–protective factor frameworks emphasizing protective processes that buffer risk links (Rutter, 1987). The present first-stage moderated mediation model moves beyond approaches that examine negative life events, self-esteem, or resilience in isolation. Specifically, it clarifies both how negative life events are associated with adolescent internalizing problems (partly via lower self-esteem) and when these associations are weaker (at higher levels of resilience). Conceptually, the findings are consistent with a self-system vulnerability account in which adversity exposure is linked to internalizing difficulties partly through diminished self-worth, while resilience functions as a protective resource that stabilizes self-related processes and is associated with weaker risk-related associations under adversity.

#### 4.4.2. Practical Implications

The findings offer potential implications for school-based mental health interventions. Given the robust association between negative life events and internalizing problems observed in the present study, educators and parents are encouraged to optimize the developmental environment. This could involve monitoring students’ stress levels and providing timely support during critical transition periods to reduce the occurrence of avoidable negative events. Furthermore, schools may benefit from complementing symptom-focused approaches with resilience-building programs. Curricula fostering a growth mindset (Yeager & Dweck, 2020), emotional regulation, and problem-solving skills may equip adolescents with the adaptive capabilities needed to navigate high-stress environments. For students exposed to high levels of negative life events, counseling interventions could benefit from prioritizing the preservation of self-esteem. Techniques that promote self-compassion and multidimensional self-identity may help prevent the internalization of external stressors into negative self-evaluations.

### 4.5. Limitations and Future Directions

Several limitations warrant consideration when interpreting the findings. First, because this study used a cross-sectional design, temporal precedence among negative life events, self-esteem, resilience, and internalizing problems cannot be firmly established. Accordingly, the findings should be interpreted as associative rather than causal, even though the proposed moderated mediation model was grounded in established theoretical frameworks. Future research should employ longitudinal or cross-lagged panel designs to clarify temporal ordering and further examine the developmental directionality of these associations.

Second, data were collected via self-report measures, which may be subject to certain biases. Specifically, since internalizing problems can carry a degree of social stigma, adolescents might have underreported their symptoms due to social desirability concerns. Although statistical checks suggested that common method variance was not a major concern, future studies might consider incorporating multi-informant data sources, such as parent reports, teacher reports, or clinical interviews. These additional sources would help enhance the validity of symptom assessment and reduce potential reporting biases.

Third, this study had limitations regarding sample representativeness and the inclusion of socio-demographic variables. Participants were recruited from public middle schools in a single metropolitan area in China using a convenience sampling method, which may limit the generalizability of the findings. Adolescents from economically developed urban areas may differ from those in rural or less-developed regions in terms of stress exposure and available support resources. Moreover, this study did not include broader socio-environmental and demographic variables, such as family structure and socioeconomic status. These variables have been shown to be associated with both stress exposure and internalizing symptoms in prior research (McLaughlin et al., 2010; Reiss, 2013). Future studies should replicate these findings across diverse geographic regions and incorporate a more comprehensive set of socio-demographic covariates to enhance external validity and better isolate the unique contributions of the key variables.

Finally, this study treated negative life events and resilience as unidimensional constructs. However, both the Adolescent Self-Rating Life Events Checklist (ASLEC; Liu et al., 1997) and the Connor–Davidson Resilience Scale (CD-RISC; Connor & Davidson, 2003) encompass multiple domains. Future research should investigate the differential buffering effects of specific resilience dimensions on various types of environmental stressors.

## 5. Conclusions

This study systematically examined the relationship between negative life events and adolescent internalizing problems by testing a moderated mediation model. Our findings indicate that negative life events are significantly associated with adolescent internalizing problems. Furthermore, self-esteem serves as an important explanatory mechanism, partially mediating this relationship by linking external environmental stressors to internal psychological distress. Moreover, psychological resilience emerges as a notable protective factor that operates through a dual protective mechanism. First, resilience directly attenuates the association between negative life events and internalizing problems, reducing adolescents’ vulnerability to stress-related emotional distress. Second, resilience safeguards the self-system by weakening the negative association between negative life events and self-esteem, thereby diminishing the indirect pathway to internalizing problems. This dual protective role of resilience is further evidenced by the finding that, at higher levels of resilience, the indirect effect via self-esteem was weakened and became non-significant.

## Figures and Tables

**Table 1:** Descriptive statistics and correlations between the main variables.

	*M*	*SD*	1	2	3	4	5	6
1 Gender	1.51	0.50	1					
2 Age	15.98	0.78	−0.06	1				
3 NLEs	41.12	11.49	0.14 **	−0.09	1			
4 IPs	5.37	5.01	0.24 **	−0.09	0.61 **	1		
5 Self-esteem	27.41	4.50	−0.05	0.03	−0.37 **	−0.54 **	1	
6 Resilience	55.32	15.06	−0.13 **	0.04	−0.25 **	−0.39 **	0.62 **	1

Notes. *N* = 400. *M* = mean; *SD* = standard deviation. NLEs = negative life events; IPs = internalizing problems. Gender was coded as 1 = male; 2 = female. ** *p* < 0.01.

**Table 2:** Decomposition of total, direct, and indirect effects of negative life events on internalizing problems via self-esteem.

	Effect	Bootstrap	95% CI		Relative Effect
		*SE*	LLCI	ULCI	
Total effect (*c*)	0.25	0.02	0.22	0.29	
Direct effect (*c*′)	0.19	0.02	0.16	0.23	76.5%
Indirect effect (*ab*)	0.06	0.01	0.04	0.08	23.5%

**Table 3:** Moderated mediation model predicting self-esteem and internalizing problems.

Predictor	Self-Esteem					IPs				
	β	*SE*	*t*	LLCI	ULCI	β	*SE*	*t*	LLCI	ULCI
Gender	0.54	0.34	1.59	−0.130	1.216	1.44	0.35	4.10 ***	0.750	2.133
Age	−0.12	0.22	−0.54	−0.542	0.310	−0.17	0.22	−0.75	−0.602	0.271
NLEs	−0.09	0.02	−6.01 ***	−0.122	−0.062	0.20	0.02	12.05 ***	0.165	0.229
Self-esteem						−0.35	0.05	−6.80 ***	−0.452	−0.249
Resilience	0.05	0.04	1.20	−0.029	0.122	0.13	0.04	3.38 ***	0.056	0.211
NLEs × Resilience	0.003	0.001	3.37 ***	0.001	0.005	−0.004	0.001	−4.01 ***	−0.006	−0.002
*R* ^2^	0.45					0.53				
Δ*R*^2^	0.02					0.02				
*F*	63.89 ***					74.29 ***				

Notes. *** *p* < 0.001. NLEs = negative life events; IPs = internalizing problems.

**Table 4:** Conditional indirect effects of negative life events on internalizing problems via self-esteem at different levels of resilience.

Moderator (Resilience)	Indirect Effect	Boot *SE*	95% Confidence Interval	
			LLCI	ULCI
*M* − 1 *SD*	0.05	0.01	0.030	0.070
*M*	0.03	0.01	0.019	0.048
*M* + 1 *SD*	0.02	0.01	−0.001	0.034

Notes. LLCI = lower limit of the 95% CI; ULCI = upper limit of the 95% CI.

## Data Availability

The datasets generated and analyzed during this study are available from the corresponding author upon reasonable request.
